# Quantification of the Active Metabolites 4‐Methylaminoantipyrine and 4‐Aminoantipyrine of Dipyrone From Human Plasma by LC–MS/MS

**DOI:** 10.1155/ianc/9944493

**Published:** 2026-04-17

**Authors:** Stefanie Schmidt, Harald Ihmsen, Tobias Golditz, Jürgen Schüttler, Andreas Wehrfritz

**Affiliations:** ^1^ Department of Anaesthesiology, University Hospital Erlangen, Friedrich-Alexander-Universität Erlangen-Nürnberg (FAU), Erlangen, Germany, fau.de

**Keywords:** 4-aminoantipyrine, 4-methylaminoantipyrine, dipyrone, liquid chromatography, mass spectrometry

## Abstract

Dipyrone (metamizole) is widely used as a nonopioid analgesic in perioperative and intensive care settings; however, its administration may be associated with severe adverse effects. In this study, we describe an analytical assay for the quantification of the active metabolites 4‐methylaminoantipyrine (4‐MAA) and 4‐aminoantipyrine (4‐AA) in human plasma using ultra‐high‐performance liquid chromatography coupled with tandem mass spectrometry (UPLC–MS/MS). In addition, the protein‐unbound fractions of both metabolites were determined by ultrafiltration. Chromatographic separation was performed on a UPLC system using gradient elution, followed by MS/MS detection with an electrospray ionization source. The limits of detection for both metabolites were 100 ng/mL. Across the investigated concentration range (100–10,000 ng/mL), the relative error (%RE) ranged from −6.3% to +3.5%. Intra‐day and inter‐assay variability were below 10%. Method validation was conducted in accordance with the 2018 FDA Bioanalytical Method Validation Guidance, and all evaluated parameters met the required criteria for analytical accuracy and precision. The developed assay is suitable for monitoring dipyrone metabolites and may support the prevention of potential overdosing during prolonged analgesic therapy or in intensive care settings.

## 1. Introduction

**FIGURE 1:**
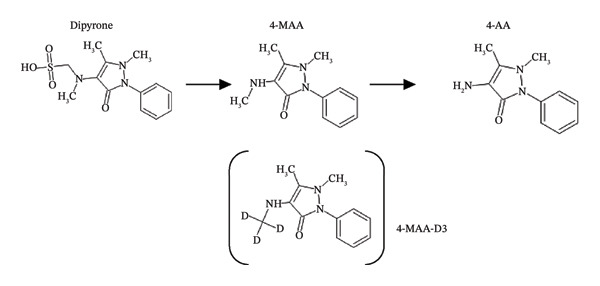
Dipyrone metabolism to the active metabolite 4‐MAA and further to 4‐AA. The internal standard deuterium labeled 4‐MAA‐D3 is shown in brackets.

Dipyrone (metamizole) is a nonopioid analgesic belonging to the pyrazolone class that is commonly used in several countries for perioperative and intensive care pain management, frequently administered as a continuous intravenous infusion. Although dipyrone is an effective analgesic, its use has been associated with potentially serious adverse effects, including agranulocytosis, hepatotoxicity, and nephrotoxicity [1, 2]. These safety concerns, combined with the frequent use of the drug in critically ill patients, highlight the importance of accurately assessing systemic drug exposure. Following oral administration, dipyrone undergoes rapid hydrolysis in the gastrointestinal tract to form its primary active metabolite, 4‐methylaminoantipyrine (4‐MAA, Figure 1). As a consequence, the parent compound becomes virtually undetectable in plasma within a few minutes [3]. A similarly rapid conversion is observed after intravenous administration [3]. The primary metabolite 4‐MAA is further metabolized by N‐demethylation, producing the secondary metabolite 4‐aminoantipyrine (4‐AA, Figure 1). The pharmacokinetics of these metabolites during prolonged intravenous administration remain incompletely characterized. In particular, it is currently unclear whether continuous infusion in humans could result in potentially toxic plasma concentrations, as has been reported in experimental studies involving dogs and other mammals [4–7]. In some intensive care units, dipyrone is administered for several days or even weeks at a fixed infusion rate without adjustment for body weight or gender. The mean plasma protein binding of 4‐MAA and 4‐AA is 57.6% and 47.9, respectively [8]. Because critically ill patients frequently exhibit alterations in protein status, such as hypoalbuminemia, changes in protein binding may occur. Under such conditions, determination of the unbound fractions of 4‐MAA and 4‐AA may therefore be particularly relevant for therapeutic drug monitoring (TDM). Only a limited number of analytical methods have been described for the quantification of dipyrone metabolites in human plasma. Earlier approaches mainly relied on high‐performance liquid chromatography (HPLC) and typically required relatively large plasma volumes [2, 8, 9]. Comparable LC–MS/MS techniques have been successfully applied in other clinical contexts, for example, in TDM in oncology patients or in metabolite profiling in preclinical studies [10, 11]. In 2016, a HPLC method capable of simultaneously determining four dipyrone metabolites was introduced. The stability of these metabolites was later reevaluated after long‐term storage of plasma samples [12]. This method already reduced the amount of sample material required for analysis. However, to date, no validated ultra‐high‐performance liquid chromatography tandem mass spectrometry (UPLC–MS/MS) method has been described for the simultaneous quantification of 4‐MAA and 4‐AA in small plasma volumes that also includes determination of the unbound fraction. This methodological gap limits the implementation of routine TDM for dipyrone. The present study therefore describes a sensitive, specific, and robust UPLC–MS/MS assay for the determination of total and unbound 4‐MAA and 4‐AA in human plasma using minimal sample volumes. The method is intended to provide the analytical basis for ongoing clinical studies and may support the future introduction of dipyrone TDM in routine intensive care practice.

## 2. Materials and Methods

The analytical procedure was established and validated in accordance with the Bioanalytical Method Validation Guidance for Industry issued by the U.S. Department of Health and Human Services Food and Drug Administration (FDA) in 2018 [13]. In addition, the methodological framework described by Eisenried et al. was taken into consideration during method development and validation [14].

### 2.1. Drugs and Chemicals

Reference standards of 4‐MAA and 4‐AA as well as the deuterated internal standard 4‐MAA‐D3 were obtained from TRC (Toronto Research Chemicals, North York, Canada). Methanol (ROTISOLV LC–MS grade), water (ROTISOLV LC–MS grade), and formic acid were purchased from Roth (Karlsruhe, Germany). Drug‐free human plasma used for preparation of calibration and validation samples (VDs) was supplied by Bio‐Rad Laboratories (Feldkirchen, Germany).

### 2.2. Internal Standard

The internal standard solution was prepared from a commercial stock solution of 4‐MAA‐D3 (1 mg/mL in methanol; TRC). For preparation of the working solution, 50 μL of the stock solution was diluted with 99.95 mL methanol. This procedure resulted in a working solution containing 500 ng/mL 4‐MAA‐D3 in methanol. The internal standard solution was subsequently added to all plasma samples during sample preparation.

### 2.3. Standard Solution and Calibration Standards

Commercial standard solutions of 4‐MAA (10 mg/mL) and 4‐AA (10 mg/mL) were combined and diluted with methanol to obtain a mixed stock solution containing both analytes at a concentration of 0.1 mg/mL. The prepared stock solution was stored at −20°C until use. Calibration samples for quantitative determination of 4‐MAA and 4‐AA were prepared by spiking drug‐free plasma with diluted standard solutions. Briefly, 50 μL of the diluted standard solution was added to 450 μL of blank plasma, resulting in final calibration concentrations of 100, 250, 500, 1000, 2500, 5000, and 10,000 ng/mL. Quality control (QC) samples were prepared in the same matrix at concentrations of 250, 1000, and 8000 ng/mL. VDs were generated using the same preparation procedure as applied for the calibration samples.

### 2.4. Sample Preparation

Prior to analysis, plasma samples were centrifuged at 2370 × g for 10 min at +4°C. For determination of the protein‐unbound fraction of 4‐MAA and 4‐AA, 150 μL plasma was transferred into a Centrifree ultrafiltration device (Merck Millipore Ltd., Carrigtwohill, Ireland). Ultrafiltration was performed to separate free analytes from plasma proteins. The devices were centrifuged at 2000 × g for 20 min at +4°C using a fixed‐angle rotor. This centrifugal force corresponded to the maximum value recommended by the manufacturer [15].

Protein precipitation was used as the extraction step for all samples. For this purpose, 50 μL of filtrate, patient sample, QC sample, or VD was mixed with 200 μL of the internal standard working solution containing 500 ng/mL 4‐MAA‐D3. After gentle vortex mixing, the samples were centrifuged again for 10 min at 2370 × g at +4°C. Following centrifugation, 150 μL of the supernatant was transferred into an HPLC vial and subjected to chromatographic analysis using the UPLC system.

### 2.5. Equipment

Quantitative analysis of the analytes was performed using UPLC–MS/MS. All instrumentation was supplied by Waters (Eschborn, Germany). Chromatographic separation was carried out on an Acquity UPLC H‐Class system equipped with a Kinetex C18 analytical column (50 × 2.1 mm, 1.7 μm particle size, 100 Å pore size; Phenomenex, Aschaffenburg, Germany). A C18 Security Guard cartridge (Waters) was used as a protective guard column. Detection was achieved with a Xevo TQD tandem mass spectrometer equipped with an electrospray ionization (ESI) interface operated in positive ion mode. Data acquisition and processing were performed using MassLynx software Version 4.2.

### 2.6. UPLC Settings

Chromatographic separation of the analytes was achieved using gradient elution. The initial mobile phase composition consisted of 5% methanol and 95% water containing 0.1% formic acid. After 0.2 min, the methanol fraction was increased to 95% and maintained for 2.3 min. Subsequently, the system was returned to the initial conditions (95% water/5% methanol, v/v) and equilibrated for 2.5 min. The total run time of the chromatographic method was 5 min. The mobile phase was delivered at a flow rate of 0.30 mL/min, and an injection volume of 2 μL was used. The column temperature was maintained at +30°C. Under these chromatographic conditions, the retention times of 4‐MAA, 4‐AA, and the internal standard 4‐MAA‐D3 were approximately 1.6 min.

### 2.7. MS/MS Settings

Mass spectrometric detection was performed using ESI in positive mode with multiple reaction monitoring (MRM). The ion source temperature was set to 150°C. Capillary voltage and cone voltage were adjusted to 3.3 kV and 31 V, respectively. The cone gas flow rate was maintained at 50 L/min. Desolvation gas temperature was set at 200°C with a gas flow rate of 650 L/min. Nitrogen served as both desolvation gas and cone gas, whereas argon was used as collision gas. The monitored ion transitions used for quantification were m/z 218.36 ⟶ 158.8 for 4‐MAA, m/z 204.01 ⟶ 55.7 for 4‐AA, and m/z 221 ⟶ 99.7 for the internal standard 4‐MAA‐D3 (Figure 2). A dwell time of 0.044 s was applied for each transition.

FIGURE 2Automatically generated positive ion mass spectrums of daughter fragment scan of 4‐MAA (a) and 4‐AA (b) by IntelliStart (Waters).

**Figure:**
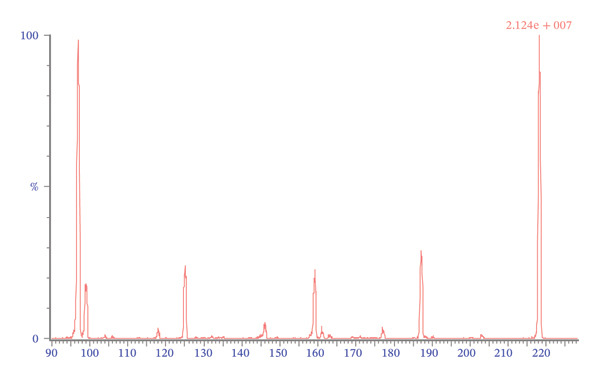
(a)

**Figure:**
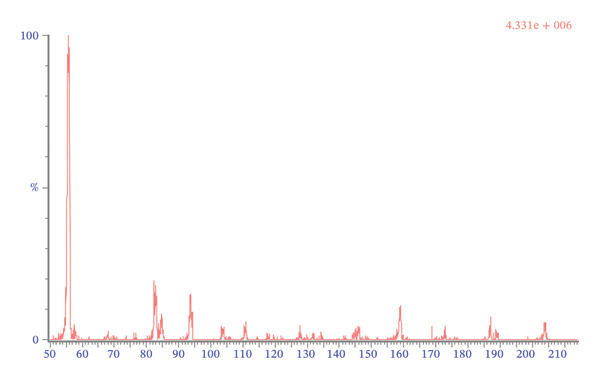
(b)

### 2.8. Method Validation

Method validation was performed in accordance with FDA recommendations for bioanalytical method validation and included evaluation of recovery, linearity, accuracy, precision, selectivity, and stability [13].

#### 2.8.1. Linearity

Linearity of the assay was assessed over three consecutive days using seven freshly prepared calibration standards covering the concentration range from 100 to 10,000 ng/mL for both analytes. Calibration curves were generated using TargetLynx software (Version 4.2; Waters). Linear regression with 1/*x* ^2^ weighting was applied. The lower limit of quantification (LLOQ) was defined as the lowest concentration on the calibration curve that fulfilled acceptance criteria of ±20% precision and 80%–120% accuracy.

#### 2.8.2. Precision and Accuracy

Intra‐assay precision and accuracy were determined by analyzing five to six replicates of each VD concentration within a single analytical run. Inter‐assay precision and accuracy were evaluated at four concentration levels (100, 250, 1000, and 10,000 ng/mL) over three consecutive days. Precision was expressed as relative standard deviation (%RSD) calculated according to %RSD = 100 × SD/M, where M represents the mean measured concentration and SD represents the corresponding standard deviation. Accuracy was expressed as relative error (%RE) calculated as %RE = 100 × (measured concentration−nominal concentration)/nominal concentration. Acceptance criteria were defined as ±15% for %RE and < 15% for %RSD.

#### 2.8.3. Assessment of Filtration Loss of 4‐MAA and 4‐AA

Potential nonspecific binding of 4‐MAA, 4‐MAA‐D3, and 4‐AA to the ultrafiltration membrane was investigated to estimate analyte loss during filtration. Standard solutions containing 4‐MAA at concentrations of 10,000, 1000, and 250 ng/mL in 0.9% NaCl were analyzed before and after filtration through the ultrafiltration devices. Both sample sets were analyzed under identical LC–MS/MS conditions. Analyte loss due to filtration was calculated using the following equation: loss (%) = ([concentration before filtration − concentration after filtration]/concentration before filtration) × 100. The overall filtration loss for each analyte was calculated as the mean value obtained from the three tested concentrations.

#### 2.8.4. Selectivity and Matrix Effect

Selectivity was evaluated using six independent lots of blank human plasma. In the first series of experiments, blank plasma samples were extracted with internal standard and analyzed directly. In the second series, plasma samples were spiked with the LLOQ concentration of 4‐MAA and 4‐AA together with the internal standard prior to extraction. Selectivity was considered acceptable if at least 90% of the analyzed blank samples showed no interfering peaks at the retention times of the analytes. Extraction recovery and matrix effects were evaluated at three concentration levels (250, 1000, and 10,000 ng/mL) with three replicates per level. VDs prepared in methanol were compared with plasma VDs processed using the extraction procedure. Matrix effects and recovery losses were calculated according to the following equation: (area plasma sample − area methanol sample)/area methanol sample × 100.

#### 2.8.5. Stability

Several stability conditions were investigated, including short‐term stability, post‐preparative stability, autosampler stability, freeze–thaw stability, and long‐term stability. For these experiments, VDs were prepared at concentrations of 100, 250, 1000, and 10,000 ng/mL. Short‐term stability was evaluated by storing samples at room temperature for 24 h prior to extraction. Post‐preparative stability was assessed by keeping processed samples after addition of internal standard at room temperature for 24 h. Autosampler stability was tested by storing samples in the autosampler at 10°C for 6 days. Freeze–thaw stability was assessed by freezing samples at −20°C for 24 h followed by thawing and analysis. This cycle was repeated three times. Long‐term stability was evaluated after storage of samples at −20°C for 93 days. According to the FDA Bioanalytical Method Validation Guidance (2018), analytes were considered stable if the measured concentrations deviated by no more than ±15% from the nominal values [13].

## 3. Results and Discussion

### 3.1. Chromatography and Mass Spectrometry

Optimal chromatographic performance was achieved using a mobile phase composed of a relatively low acid concentration combined with a high proportion of organic solvent. To ensure compatibility with ESI, analytes were initially dissolved in methanol prior to injection. During chromatographic separation, the proportion of methanol was gradually increased to 95%. The ionic strength and pH of the mobile phase were stabilized by the addition of formic acid and ammonium acetate to all eluents. A constant flow rate of 0.3 mL/min was maintained throughout the analytical run and provided satisfactory chromatographic resolution without compromising peak shape or analytical performance. Mass spectrometric parameters were optimized to maximize the signal intensity of protonated molecular ions ([M+H]+). Operation in positive ESI mode produced abundant fragment ions suitable for MRM. The selected ion transitions were m/z 218.03 ⟶ 158.83 for 4‐MAA, m/z 221 ⟶ 99.7 for the internal standard 4‐MAA‐D3, and m/z 204.01 ⟶ 55.72 for 4‐AA. No interference from endogenous plasma components or other metabolites was observed at the retention times of the analytes when blank plasma samples were analyzed. All analytes (4‐MAA, 4‐AA, and 4‐MAA‐D3) eluted at approximately 1.6 min. Although coelution of analytes is generally avoided in classical chromatographic methods, such overlap can be tolerated in LC–MS/MS when sufficient specificity is provided by the mass spectrometer. In the present method, compound‐specific MRM transitions ensured analytical selectivity independently of chromatographic separation. Possible cross‐talk between transitions and ion suppression effects were evaluated during method validation and were not detected.

### 3.2. Linearity and Limit of Detection (LOD) and Quantification

The calibration curves of 4‐MAA and 4‐AA were linear within the studied range from 100 to 10,000 ng/mL (*y* = 0.00246 ∗ *x* + 0.220, *r* ^2^ = 0.999 and *y* = 0.0537 ∗ *x* + 7.708, *r* ^2^ = 0.999, respectively).

The LOD of both 4‐MAA and 4‐AA was 1 ng/mL with a signal‐to‐noise ratio (S/N ratio) of 254 and 490, respectively. Figure 3 shows typical chromatograms of plasma spiked with the LOD concentration of 4‐MAA and 4‐AA. The LLOQ for both metabolites was 100 ng/mL.

FIGURE 3Representative chromatogram of plasma spiked with the limit of detection (LOD) concentration of 4‐MAA (a) and 4‐AA (b). LOD was 1 ng/mL with a signal‐to‐noise ratio (S/N) of 254 and 490, respectively.

**Figure:**
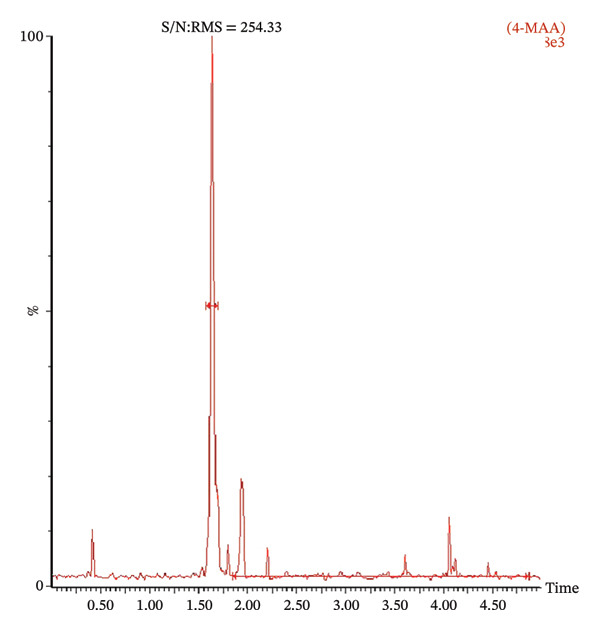
(a)

**Figure:**
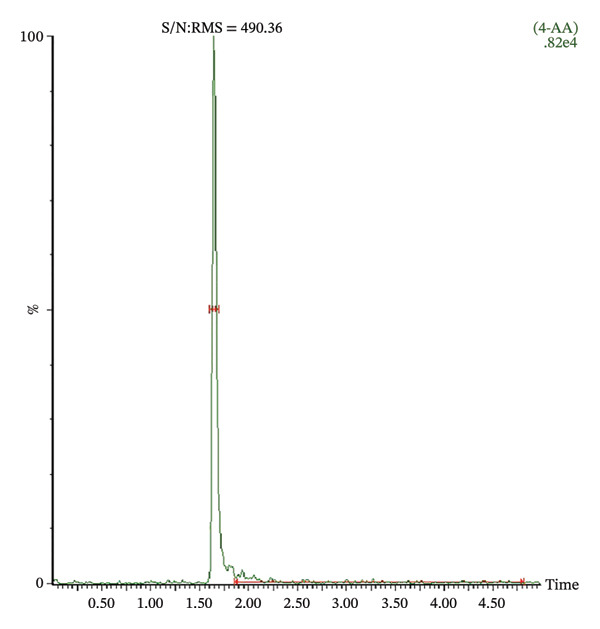
(b)

### 3.3. Precision and Accuracy

Intra‐day and inter‐day precision and accuracy of both compounds were good (Table 1). Even the largest deviation with an inter‐assay RE of −6.3% for 4‐MAA at 10,000 ng/mL was clearly within the regulatory limits of acceptance (±15%).

**TABLE 1:** Accuracy and precision of the analytical method for the quantification of 4‐MAA and 4‐AA in human plasma.

Analyte	Nominal concentration (ng/mL)	Intra‐day				Inter‐assay			
		Mean ± SD	%RSD	%RE	*n*	Mean ± SD	%RSD	%RE	*n*
4‐MAA	100	100 ± 6.1	6.1	0.3	6	100 ± 5.5	5.5	0.1	16
	250	251 ± 10.1	4.0	0.4	5	252 ± 10.9	4.3	1.1	15
	1000	981 ± 16.3	1.7	−1.9	5	966 ± 26.6	2.8	−3.4	15
	10,000	9591 ± 179	1.9	−4.1	5	9374 ± 226	2.4	−6.3	15

4‐AA	100	97.7 ± 4.0	4.1	−2.3	6	101 ± 4.3	4.3	0.6	16
	250	255 ± 7.4	2.9	1.9	5	259 ± 6.4	2.5	3.5	15
	1000	990 ± 17.4	1.8	−1.0	5	1003 ± 21.6	2.2	0.3	15
	10,000	9610 ± 164	1.7	−3.9	5	9665 ± 182	1.9	−3.4	15

*Note:* % RSD, relative standard deviation; % RE, relative error from nominal concentration.

### 3.4. Sensitivity and Selectivity

Matrix effects were evaluated at concentrations of 250, 1000, and 10,000 ng/mL. Method selectivity was assessed by comparing chromatograms obtained from blank patient plasma with those from drug‐free plasma samples spiked with the analytes as well as from untreated patient plasma samples (Figure 4). None of the six tested plasma batches produced significant interfering signals at the retention times of the analytes or the internal standard. The ratio of signal response between blank interference and the LLOQ standard exceeded a factor of 50 for all investigated compounds. These findings demonstrate that the analytical method provides adequate selectivity and sensitivity for reliable detection of both metabolites in human plasma.

FIGURE 4Chromatogram of a blank sample (a), a zero sample with internal standard 4‐MAA‐D3 (b) and a blank plasma sample mixed with 4‐MAA/4‐AA in a concentration of 100 ng/mL (c).

**Figure:**
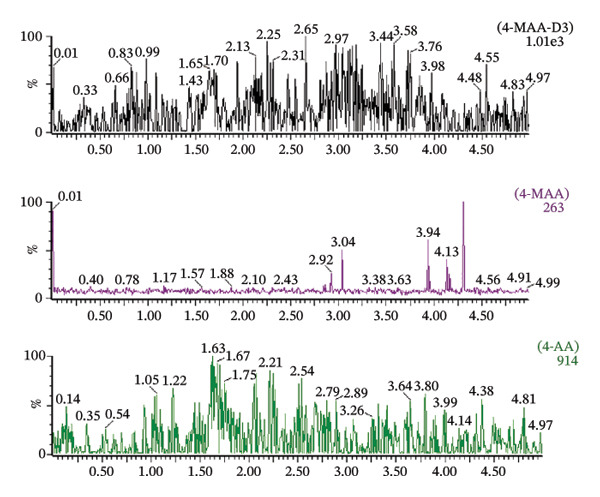
(a)

**Figure:**
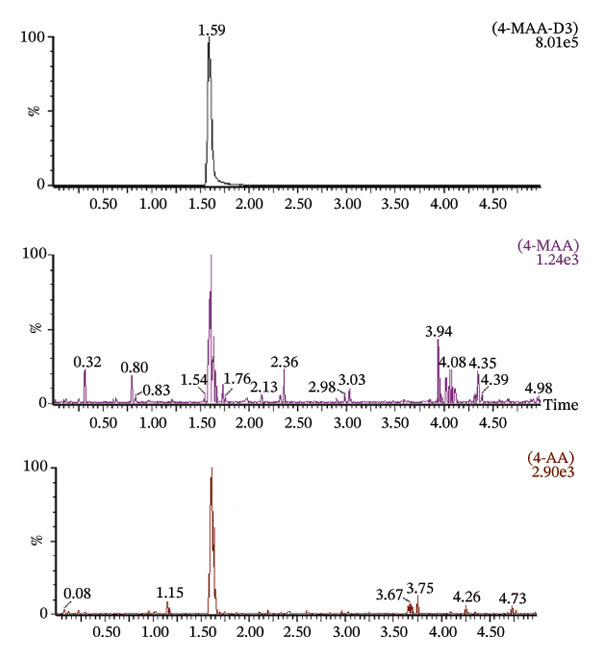
(b)

**Figure:**
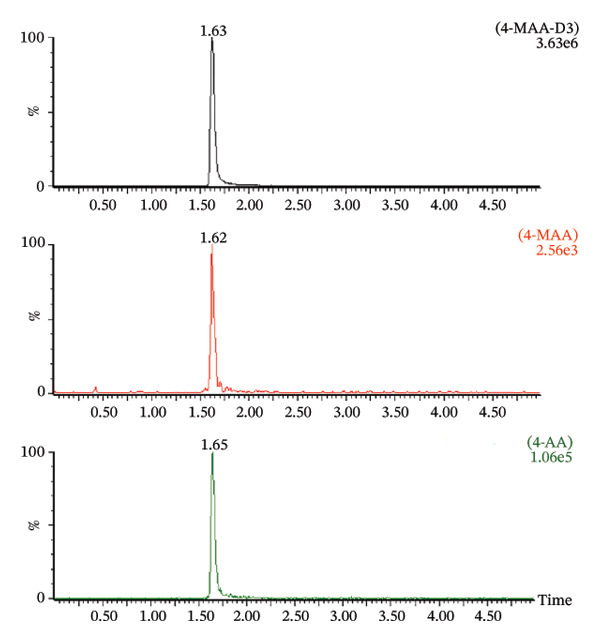
(c)

### 3.5. Loss of Analytes During Ultrafiltration

The ultrafiltration procedure used to separate the unbound drug fraction was evaluated with respect to potential analyte losses (Table 2). For 4‐MAA, a pronounced loss was observed at the lowest tested concentration (250 ng/mL), where the mean loss amounted to 18.3 ± 7.7%. In contrast, negligible losses were observed at higher concentrations (1000 and 10,000 ng/mL). Slightly negative values obtained at these concentrations most likely reflect normal analytical variability rather than an actual increase in analyte concentration. When averaged across all concentration levels, the overall mean loss of 4‐MAA was calculated as 5.6 ± 9.0%. For 4‐AA, analyte losses were generally lower and showed less variability across the investigated concentration range. The overall mean loss for this compound was 6.1 ± 3.8%, which is considered acceptable for bioanalytical assays.

**TABLE 2:** Loss of analytes during ultrafiltration for free fraction separation.

Analyte	Nominal concentration (ng/mL)	Loss (%, mean ± SD)
4‐MAA	250	18.3 ± 7.7
	1000	−0.5 ± 2.7
	10,000	−1.0 ± 2.8
	Mean	5.6 ± 9.0

4‐AA	250	10.7 ± 4.0
	1000	6.3 ± 2.1
	10,000	1.4 ± 0.6
	Mean	6.1 ± 3.8

These observations indicate that ultrafiltration is a suitable technique for determining the unbound fraction of both analytes. However, a concentration‐dependent bias was observed, particularly for 4‐MAA at low concentrations. This effect should be taken into account when interpreting free‐fraction data. In cases involving very low analyte concentrations, correction strategies or alternative separation approaches may be required to ensure accurate quantification.

### 3.6. Stability

Stability experiments demonstrated that both analytes remained stable under all tested conditions. No relevant degradation of 4‐MAA or 4‐AA was observed during post‐extraction storage (48 h), autosampler storage at 10°C for six days, three freeze–thaw cycles, or long‐term storage at −20°C for 93 days (Table 3). In all experiments, measured concentrations deviated by less than 15% from nominal values, fulfilling regulatory acceptance criteria. In addition, the stock solutions of the analytes remained stable during short‐term storage at room temperature for 24 h. These findings indicate that the analytical procedure is suitable for routine laboratory applications without relevant stability limitations.

**TABLE 3:** Stability analysis of the analytical method for the measurement of 4‐MAA and 4‐AA in human plasma.

	***n***	**4-MAA**			**4-AA**		
		**Mean ± SD (%RE)**			**Mean ± SD (%RE)**		
		**10,000 ng/mL**	**1000 ng/mL**	**250 ng/mL**	**10,000 ng/mL**	**1000 ng/mL**	**250 ng/mL**

Short term^a^	6	9213 ± 503 (−7.9)	942 ± 55.3 (−5.8)	248 ± 14.4 (−1.0)	9699 ± 261 (−3.0)	990 ± 20.4 (−1.0)	259 ± 2.1 (3.5)
Long term^b^	6	9617 ± 150 (−3.8)	980 ± 16.0 (−2.0)	252 ± 11.7 (0.9)	9779 ± 224 (−2.2)	994 ± 30.3 (−0.6)	257 ± 5.6 (3.0)
Freeze‐thaw stability^c^	12	9505 ± 265 (−5.0)	986 ± 35.8 (−1.4)	259 ± 6.2 (3.4)	9707 ± 213 (−2.9)	997 ± 23.0 (−0.4)	260 ± 4.1 (3.9)
Autosampler stability^d^	12	9514 ± 198 (−4.9)	992 ± 30 (−0.8)	253 ± 8.3 (1.3)	9682 ± 219 (−3.2)	944 ± 21.4 (−0.6)	259 ± 4.6 (3.1)
Post‐extraction stability^e^	6	9641 ± 180 (−3.6)	990 ± 28.4 (−1.0)	254 ± 5.5 (2.6)	9713 ± 219 (−2.9)	1000 ± 23.7 (−0.05)	257 ± 5.5 (2.6)

*Note:* %RE, relative error from nominal concentration.

^a^Room temperature for 24 h after thawing.

^b^Stored at −20°C for 93 days.

^c^After three freeze and thaw cycles.

^d^Stored in autosampler for 6 days at 10°C.

^e^Stored 24 h at room temperature after extraction.

### 3.7. Comparison With Previous Studies

Only a limited number of analytical methods have been reported for the quantification of dipyrone metabolites in human plasma following intravenous administration using UPLC–MS/MS with MRM. Bachmann et al. [12] described a validated analytical method capable of quantifying all four principal metabolites of dipyrone from small plasma volumes. The analytical performance of the present method is largely comparable to that previously reported. However, while the method by Bachmann et al. relied on conventional HPLC conditions with a flow rate of 0.7 mL/min, the present approach utilizes UPLC conditions with a reduced flow rate of 0.3 mL/min. This reduction results in lower solvent consumption and improved analytical efficiency while maintaining comparable analytical reliability. Although the overall analytical performance is similar, the reduced solvent usage and compatibility with modern UPLC instrumentation represent practical advantages for routine bioanalytical applications. To the best of our knowledge, the only previously published method for determining the unbound (protein‐free) fractions of 4‐MAA and 4‐AA in plasma was reported by Zylber‐Katz et al. in 1985 [8], which required plasma volumes of approximately 2–3 mL. Subsequent studies mainly focused on total plasma concentrations and pharmacokinetic analyses of these metabolites, without addressing the measurement of the unbound fraction. The analytical procedure described here addresses this methodological gap by enabling reliable quantification of the free fraction using only 150 μL of plasma. This substantial reduction in required sample volume represents a significant advantage compared with earlier approaches and facilitates application in contemporary clinical and preclinical pharmacokinetic studies.

## 4. Conclusion

The present method provides a reliable and sensitive assay for the measurement of the total and unbound plasma concentrations of both dipyrone metabolites 4‐MAA and 4‐AA in a small amount of human plasma. All validation parameters were within the regulatory limits. The method requires only 50 μL of sample for total concentrations and 150 μL for the determination of unbound concentrations, making it suitable for measuring plasma levels during and after surgery as well as for drug monitoring in intensive care units, where minimizing blood loss is crucial in functionally anemic postoperative patients. The assay provides the methodological basis for future clinical investigations aimed at characterizing plasma concentrations during prolonged intravenous infusion, exploring interindividual variability, and evaluating potential links between drug exposure and adverse effects.

## Author Contributions

Stefanie Schmidt: conceptualization, investigation, formal analysis, methodology, writing–original draft, and writing–review and editing.

Harald Ihmsen: conceptualization, formal analysis, writing–original draft, and writing–review and editing.

Tobias Golditz: conceptualization, investigation, and writing–review and editing.

Jürgen Schüttler: conceptualization and writing–review and editing.

Andreas Wehrfritz: conceptualization, investigation, formal analysis, writing–original draft, and writing–review and editing.

## Funding

This research did not receive any specific grant from funding agencies in the public, commercial, or not‐for‐profit sectors. Open Access funding enabled and organized by Projekt DEAL.

## Disclosure

The present work was performed in (partial) fulfillment of the requirements for obtaining the degree “Dr. rer. biol. hum.” at the Friedrich‐Alexander‐Universität Erlangen‐Nürnberg (FAU).

## Conflicts of Interest

The authors declare no conflicts of interest.

## Data Availability

The data supporting the results of this study are available from the corresponding author upon request.

## References

[1] Lutz M. , Metamizole (Dipyrone) and the Liver: A Review of the Literature, Journal of Clinical Pharmacology. (2019) 59, no. 11, 1433–1442, 10.1002/jcph.1512, 2-s2.0-85070898636. 31433499

[2] Hinz B. , Cheremina O. , Bachmakov J. et al., Dipyrone Elicits Substantial Inhibition of Peripheral Cyclooxygenases in Humans: New Insights Into the Pharmacology of an Old Analgesic, FASEB Journal. (2007) 21, no. 10, 2343–2351, 10.1096/fj.06-8061com, 2-s2.0-34547772738. 17435173

[3] Levy M. , Zylber-Katz E. , and Rosenkranz B. , Clinical Pharmacokinetics of Dipyrone and Its Metabolites, Clinical Pharmacokinetics. (1995) 28, no. 3, 216–234, 10.2165/00003088-199528030-00004, 2-s2.0-0028918176. 7758252

[4] Mouta A. N. , Arcoverde K. N. , Fernandes N. S. et al., Pharmacokinetic Profile of Two Active Dipyrone Metabolites, 4-Methylaminoantipyrine (MAA) and 4-Aminoantipyrine (AA), Following Intravenous Administration in Dogs: A Preliminary Study, Animals. (2025) 15, no. 11, 10.3390/ani15111666. PMC12153896 40509132

[5] Kim T. W. , Łebkowska‐Wieruszewska B. , Sitovs A. et al., Pharmacokinetic Profiles of Metamizole (Dipyrone) Active Metabolites in Goats and Its Residues in Milk, Journal of Veterinary Pharmacology and Therapeutics. (2018) 41, no. 5, 699–705, 10.1111/jvp.12679, 2-s2.0-85052207219. 29943417

[6] O′Banion M. P. , Sundman E. , Edmonds M. , and Davis J. , Pharmacokinetics of Dipyrone in Horses: A Multi‐Dose, Dose Escalation Study, Journal of Veterinary Pharmacology and Therapeutics. (2021) 44, no. 6, 919–926, 10.1111/jvp.12996. 34228836

[7] Arcoverde K. N. , Alves LdS. , Cavalcante J. M. et al., Pharmacotherapeutic Monitoring of Dipyrone in Northeastern Brazilian Donkeys (*Equus asinus*), Research in Veterinary Science. (2023) 164, 10.1016/j.rvsc.2023.105034. 37820460

[8] Zylber-Katz E. , Granit L. , and Levy M. , Plasma Protein Binding of Dipyrone Metabolites in Man, European Journal of Clinical Pharmacology. (1985) 29, no. 1, 67–71, 10.1007/bf00547371, 2-s2.0-0021933136. 4054207

[9] Ojha A. , Rathod R. , and Padh H. , Quantification of 4-Methylaminoantipyrine, the Active Metabolite of Dipyrone, in Human Plasma, Bioanalysis. (2009) 1, no. 2, 293–298, 10.4155/bio.09.26, 2-s2.0-79957966040. 21083168

[10] Lou Y. , Sun Z. , Chai Y. et al., Simultaneous Quantification of Donafenib, Sorafenib, and Their N-Oxide Metabolites in Rat Plasma Using a HPLC-MS/MS Method, Journal of Chromatography B. (2023) 1229, 10.1016/j.jchromb.2023.123871. 37717473

[11] Lou Y. , Cheng M. , Cao Q. et al., Simultaneous Quantification of Mirabegron and Vibegron in Human Plasma by HPLC-MS/MS and Its Application in the Clinical Determination in Patients With Tumors Associated With Overactive Bladder, Journal of Pharmaceutical and Biomedical Analysis. (2024) 240, 10.1016/j.jpba.2023.115937. 38198885

[12] Bachmann F. , Blaser L. , Haschke M. , Krähenbühl S. , and Duthaler U. , Development and Validation of an LC–MS/MS Method for the Bioanalysis of the Major Metamizole Metabolites in Human Plasma, Bioanalysis. (2020) 12, no. 3, 175–189, 10.4155/bio-2019-0251. 32052638

[13] Bioanalytical Method Validation. Guidance for Industry, 2018, U.S. Food and Drug Administration.

[14] Eisenried A. , Peter J. , Lerch M. , Schüttler J. , and Jeleazcov C. , HPLC-MS/MS Based Time Course Analysis of Morphine and Morphine-6-Glucoronide in ICU Patients, Journal of Chromatography & Separation Techniques. (2017) 08, no. 03, 10.4172/2157-7064.1000368.

[15] Sigma-Aldrich , Centrifree® Ultrafiltration Device User Guide, 2024.

